# Lifting the curse of knowing: How feedback improves perspective-taking

**DOI:** 10.1177/1747021820987080

**Published:** 2021-02-04

**Authors:** Debby Damen, Marije van Amelsvoort, Per van der Wijst, Monique Pollmann, Emiel Krahmer

**Affiliations:** Department of Communication and Cognition, Tilburg School of Humanities and Digital Sciences, Tilburg University, Tilburg, The Netherlands

**Keywords:** Perspective-taking, egocentric bias, curse of knowledge, egocentric projection, feedback

## Abstract

People are likely to use their own knowledge as a frame of reference when they try to assess another person’s perspective. Due to this egocentric anchoring, people often overestimate the extent to which others share their point of view. This study investigated which type of feedback (if any) stimulates perceivers to make estimations of another person’s perspective that are less biased by egocentric knowledge. We allocated participants to one of the three feedback conditions (no feedback, accuracy feedback, narrative feedback). Findings showed that participants who were given feedback adjusted their perspective-judgement more than those who did not receive feedback. They also showed less egocentric projection on future assessments. Participants adjusted their perspective within the same trial to the same degree for both feedback types. However, participants’ egocentric bias was only reduced when they received narrative feedback and not when they received accuracy feedback about their performance. Implications of these findings for theories of perspective-taking are discussed.

## Introduction

In our everyday life, we often try to infer other people’s inner motives, thoughts, and feelings by observing their behaviour. We may interpret a helping hand as a genuine display of kindness or a sad face of a child opening a present as a signal of disappointment. When we try to interpret other people’s mental states, we engage in so-called *perspective-taking*. During perspective-taking, we often rely on our own thought processes to understand those of others. This reliance on our own knowledge, however, might lead to an overreliance on the self-perspective, leading to an overestimation of the similarity between our own and the other’s point of view (e.g., Epley & Gilovich, 2001, 2006; Epley et al., 2004, 2006; Keysar, 1994). In this study, we examine what people need to overcome this overreliance on the self-perspective.

One prevalent question in the perspective-taking research is whether and how people acknowledge others’ perspectives in communication. The theoretical accounts that address this question differ in whether and how communicators integrate their interlocutor’s perspective from the onset of language processing. For instance, constrain-based accounts argue that people use their own and others’ perspectives simultaneously, depending on the discourse context (see Brown-Schmidt & Hanna, 2011; Heller et al., 2016, for a discussion) or on their ability to suppress an egocentric representation (e.g., Barr, 2008; Brown-Schmidt, 2009). Indeed, studies have shown that interlocutors are able to rapidly integrate their interlocutor’s perspective in the early stages of language production and understanding (e.g., Brown-Schmidt et al., 2008; Brown-Schmidt & Hanna, 2011; Brown-Schmidt & Tanenhaus, 2008; Hanna & Tanenhaus, 2004; Hanna et al., 2003; Heller et al., 2008, 2012; Nadig & Sedivy, 2002) and to switch between representing their own and others’ perspectives in a swift manner (e.g., Apperly et al., 2010; Ferguson et al., 2017; Samson et al., 2010; Samuel et al., 2019). These findings suggest that interlocutors switch between perspective-taking strategies, depending on the circumstances of the discourse. A different theoretical account is the egocentric anchoring and adjustment framework, postulating that communicators use their egocentric representations as a default to infer those of their interlocutor, integrating their interlocutor’s perspective in a later, optional stage when egocentric errors need to be adjusted for (Damen et al., 2020; Epley et al., 2004; Horton & Keysar, 1996; Keysar et al., 1998, 2000, 2003). In these instances, interlocutors overestimate the extent to which their egocentric point of view is accessible (or transparent) to others. The focus of this article is not on providing evidence for one account over the other. Our aim is to examine in more detail those instances in which egocentric errors occur. We investigate what it takes for communicators to rely less on their egocentric perspective and how feedback helps in this process.

### The self-perspective in social perception

Perceivers’ overestimation of the similarity between their own and the others’ perspective has been documented in many different contexts, including decision-making (Bazerman & Neale, 1982; Camerer et al., 1989; Garcia, 2002; Van Boven et al., 2000), language production (e.g., Damen et al., 2018; Wardlow Lane & Liersch, 2016; Wardlow Lane et al., 2006), and language comprehension processes (e.g., Epley et al., 2004; Gerrig et al., 2000; Keysar, 1994; Weingartner et al., 2009; Weingartner & Klin, 2005). In the latter case, studies have shown that readers are likely to overestimate the extent to which their own knowledge about a story character’s intention is accessible to a less informed protagonist. A classic example is Epley et al.’s (2004) “Sarcastic Messages” experiment. In their study, participants read stories in which a speaker left an ambiguous voicemail message (e.g., “About that dancing class: I can’t think of better ways to spend my Tuesday evenings”) on the answering machine of an addressee. The voicemail was ambiguous in the sense that it could be interpreted as either sarcastic or sincere. Participants (the readers) received clarifying event information before they listened to the voicemail and, therefore, knew whether it was sarcastic or sincere. For example, participants read that the speaker thought the dance class had been dull, causing the speaker’s message to be interpreted as sarcastic by readers themselves. The addressee did not receive disambiguating information and, hence, had no reason to believe that the speaker intended his voicemail to be sarcastic. Epley et al. (2004) showed that readers nevertheless used the clarifying information and assumed that the addressee would also perceive the speaker’s sarcasm. Once readers had resolved the message’s ambiguity, they were unable to appreciate that the speaker’s intention was less clear to the addressee than it was to readers themselves (see also the illusory transparency of intention in Keysar, 1994, 2000, and curse of knowledge effect in Camerer et al., 1989).

Epley et al. (2004) argued that readers’ perspective-taking process followed two sequential phases: an egocentric anchoring phase and an adjustment phase (see also Barr & Keysar, 2005; Epley, 2008; Epley & Gilovich, 2004; Jacowitz & Kahneman, 1995; Kahneman, 2011; Quattrone, 1982; Tversky & Kahneman, 1974). During the first phase, readers use their own interpretation of the speaker’s voicemail as a frame of reference (egocentric anchor) when they try to determine the addressee’s interpretation. Subsequently, readers adjust away from this egocentric anchor to account for possible informational differences between their own and the addressee’s perception. Because readers know the speaker’s actual intention, adjustments away from this knowledge are often insufficient (see also Epley & Gilovich, 2004; Gilovich & Epley, 2006; Jacowitz & Kahneman, 1995; Quattrone, 1982; Tversky & Kahneman, 1974). The resulting judgement, therefore, is likely to reflect too much of readers’ own interpretation of the speaker’s message. By comparing two groups of readers, Epley et al. (2004) found evidence for this egocentric anchoring and adjustment during perspective-taking. One group of readers was instructed only to indicate their own perception of the speaker’s sarcasm, whereas the other group only judged addressees’ perception of the speaker’s sarcasm. Findings showed that the perception of sarcasm was larger in the first group than in the second. Therefore, readers did acknowledge that the messages were likely to be perceived as more ambiguous by the uninformed addressees than by themselves and adjusted their own interpretation. However, readers in the second group still overestimated the extent to which the addressee would perceive the speaker’s sarcasm, showing that the perspective-adjustments were not sufficient to reflect addressees’ true perspective. In other words, readers’ knowledge about the speaker’s intention still “cursed” their own interpretation of the message (Birch & Bloom, 2007; see also Camerer et al., 1989). This raises the question of what readers might need in those instances to adjust away from an egocentric interpretation and to form a more accurate judgement. In this article, we will examine whether and which type of feedback helps in this process.

### Adjusting the self-perspective

Various studies have proposed that perceivers’ egocentric reasoning might be caused by their inability to suppress salient (e.g., Bayen et al., 2007; Lagattuta et al., 2010), highly accessible knowledge (e.g., Harley et al., 2004; see Birch et al., 2017, for a review). Building on these mechanisms, several studies proposed factors that might decrease egocentric projection. For instance, it is known that perceivers are more likely to appreciate another person’s vantage point if they are able to inhibit their egocentric viewpoint (e.g., Samuel et al., 2019), when they exert motivational and cognitive resources (e.g., Cane et al., 2017; Epley & Gilovich, 2004, 2006; Epley et al., 2004, 2006; Ferguson et al., 2017; Simmons et al., 2010), when they reason counterfactually (e.g., Pohl, 1998; see also Walker Naylor et al., 2011), or when they are aware of their biased interpretations (e.g., Bazerman & Neale, 1982; Gilovich & Epley, 2006). Following this line of reasoning, Damen et al. (2020) examined whether perceivers would rely less on their privileged perspective when they were explicitly instructed to attend to another person’s knowledge and attentional status. They tested this assumption in a direct replication and extension of Epley et al.’s (2004) “Sarcastic Messages” experiment. In Damen et al. (2020), participants were asked to indicate which information about the speaker’s experience was known to addressees before they estimated addressees’ interpretation of the voicemail. Importantly, when participants answered that addressees knew about the speaker’s experience, participants were informed that their answer was wrong and that addressees did not have access to this clarifying event information.^1^ In addition to replicating the general findings of Epley et al. (2004), Damen et al. (2020) showed that participants still thought addressees would perceive the speaker’s sarcasm, regardless of an explicit focus on addressees’ uninformed perspective. In this sense, explicit instructions to focus their attention on another person’s perspective did not help perceivers to inhibit their privileged perspective to increase their perspective-taking accuracy. These findings by Damen et al. (2020) are in line with related studies (Damen, van Amelsvoort, et al., 2019; Damen, van der Wijst, et al., 2019), evidencing that an explicit focus on or an explicit awareness of another person’s different perspective does not suffice to reduce perceivers’ tendency to overrely on their own knowledge and attentional status during perspective-taking. It is therefore still unknown how perceivers can be stimulated to sufficiently adjust the self-perspective. In Damen et al. (2020), participants never learned that they misattributed their perception of the speaker’s sarcasm onto addressees. Here, we address the question of whether this information of misattribution might help perceivers to improve the accuracy of their perspective-taking judgements.

### The role of feedback in debiasing social judgements

It is reasonable to assume feedback might help perceivers to engage in more accurate perspective-taking by updating their mental representation and engaging in more elaborative thinking to reduce possible biases (e.g., Creyer & Ross, 1993; Petty et al., 1981). Many studies have shown that feedback helps both in everyday conversation (Horton & Gerrig, 2002; Krauss & Fussell, 1991; Krauss et al., 1977; Krauss & Weinheimer, 1966; Kraut et al., 1982; Matthews et al., 2007; Wardlow & Heyman, 2016) and in training for children and clinical groups (Bechi et al., 2012; Wellman et al., 2002). However, other studies highlight significant problems with the generalisability of these effects to other tasks and everyday mindreading (Begeer et al., 2011), and poor maintenance of these trained skills over time (e.g., Williams et al., 2012). To identify their commonalities and differences, it is important to regard the content of the feedback that is provided to perceivers. Outcome or performance feedback communicates the correctness of the judgements to individuals immediately after they have made a mistake (e.g., Balzer et al., 1989). This type of feedback is proven most effective in predictable, simple tasks in which judgers are able to trace back what resulted in them making this mistake (e.g., Hirst et al., 1999). Based on previous literature, we might expect that it is quite difficult to debias social judgement by means of performance feedback. For example, people still overestimate the similarity between their own and others’ perspectives after receiving feedback about the accuracy of their responses (Camerer et al., 1989). Previous findings do suggest, however, that repeated experience (e.g., Camerer et al., 1989; West, 1996) or providing perceivers with detailed, individuated information about others’ preferences (e.g., Eyal et al., 2018; Thompson & DeHarpport, 1994) might stimulate interpersonal accuracy. Note that the situations in which the efficacy of this performance feedback was tested were non-ambiguous, with perceivers learning to predict a person’s trading preferences (Camerer et al., 1989), their preference for quilt patterns (West, 1996), negotiation interests (Thompson & DeHarpport, 1994), or opinions (e.g., Eyal et al., 2018). We wonder whether performance feedback can stimulate interpersonal accuracy in a communicative context that is by definition ambiguous.

A study by Weingartner and Klin (2005) suggests that presenting perceivers with accurate, detailed information about another person’s uptake of an ambiguous message allows perceivers to recognise that the other person’s perspective is different from their own. Weingartner and Klin (2005) tracked participants’ reading time while they read a description of a story character’s perspective that was different from the participants’ self-perspective. To illustrate this, imagine that participants read the Dance Class scenario we previously described in this introduction. Recall that, in this scenario, the speaker (Tom) attends a dance lesson and he informs his friend Eileen (the addressee) about his experience (i.e., “I cannot think of better ways to spend my Tuesday evenings”). As participants had previously learned that Tom did not enjoy the dance lesson, they can be expected to interpret Tom’s message as being sarcastic. In Weingartner and Klin (2005), participants were subsequently presented with Eileen’s sincere interpretation of Tom’s note (e.g., the target line: “Eileen could not wait to join Tom in the dance lesson”). Weingartner and Klin (2005) showed that the reading times on this target line were longer when participants’ privileged information suggested that Tom was being sarcastic than when participants’ privileged information suggested Tom was being sincere. The authors argued that this slowdown in reading demonstrated that participants spend some time processing that Eileen’s knowledge was different from their own perspective. Unfortunately, Weingartner and Klin (2005) did not examine whether this online processing of another person’s (different) perspective stimulated participants to adjust their judgement of Eileen’s perspective. Therefore, it remains unexplored whether feedback helps perceivers to acknowledge this information in their perspective-judgement and whether this feedback decreases perceivers’ egocentric projection on future perspective-taking attempts.

### Performance feedback: accuracy and narrative feedback

In this study, we explore the extent to which performance feedback helps perceivers to adjust their judgement in later perspective-taking attempts and which type of feedback works best. Feedback can either be explicit, by informing participants about the accuracy of their judgement and how they can improve it (e.g., Camerer et al., 1989; West, 1996), or be implicit by giving participants information about the addressee that allows them to assess their accuracy (e.g., Eyal et al., 2018; Thompson & DeHarpport, 1994; Weingartner & Klin, 2005). To point out these differences and their assumed efficacy in increasing perceivers’ interpersonal accuracy, we build on the distinction that is made in language learning research on “corrective feedback”(e.g., Ellis et al., 2006). Corrective feedback can take either an explicit form (“No, she did not *go-ed*—she went”) or an implicit form (e.g., “She went”) (Ellis et al., 2006; see also indirect vs direct feedback in Van Beuningen et al., 2008). In the explicit form, the error is identified and learners are provided with information about how to correct their mistake. By contrast, implicit feedback takes the form of recasting the utterance of the learner in a correct format, without explicitly signalling the error. In this case, the nature of the error often remains unclear and learners have to infer by themselves that an error has been made.

Although receiving implicit feedback might stimulate a more reflective learning process because learners need to exert more effort in self-editing their errors (e.g., Ferris, 1995), the danger of receiving this type of feedback is that learners might not be aware of its corrective intent (e.g., Nicholas et al., 2001) or that they do not know how to improve their accuracy (e.g., Chandler, 2003; Van Beuningen et al., 2008). Studies indeed evidenced the advantage of explicit feedback over implicit feedback on accuracy, with regard to both short-term (Ellis et al., 2006) and long-term learning effects (Chandler, 2003; Van Beuningen et al., 2008). In addition, studies have shown that learners were more likely to perceive the feedback as corrective when it was explicit rather than implicit (Ellis et al., 2006). Hence, when given explicit feedback about their performance, learners were more aware that they needed to improve their learning. We thus expect that the explicitness of the performance feedback might affect whether and how well perceivers learn to take perspective. To our knowledge, the efficacy of these different forms of performance feedback on perceivers’ perspective-taking has not yet been examined. In this study, we will test the extent to which these different feedback types affect perceivers’ ability to arrive at an accurate interpersonal understanding.

## Current study

This study examines the role of performance feedback as a means to gain accurate insight into another person’s perspective. In two experiments, we investigate whether confronting readers with feedback helps them to better re-assess addressees’ perspective, decreasing the tendency to attribute their perception of a speaker’s sarcasm onto these addressees. Experiment 1 explores whether readers adjust their perspective differently depending on which type of feedback they receive. They receive either explicit feedback about the correctness of the response (we will call this *accuracy feedback*) or implicit feedback about the correctness of their perspective-judgement by showing the interpretation of the addressee (we will call this *narrative feedback*). The narrative feedback contains detailed information about addressees’ perspective and the story development, but the participant is not explicitly told to what extent their responses are accurate (e.g., Weingartner & Klin, 2005). We assess perceivers’ perspective-adjustments by asking them to judge addressees’ perception twice for each story, before and after receiving feedback. In Experiment 2, we aim to replicate the findings of Experiment 1. Moreover, we let readers judge addressees’ perspective only once for each story, to test whether possible perspective-adjustment effects demonstrated in Experiment 1 occurred due to the feedback readers received and not due to them assessing addressees’ perspective twice for each story.

## Experiment 1

This study replicates and extends Damen et al.’s (2020) study in which readers judged addressees’ interpretation of voicemails sent by a speaker protagonist. We extend the experimental design by adding a feedback manipulation and a subsequent second measurement of readers’ judgement of addressees’ interpretation of the voicemail. In line with previous findings (Damen et al., 2020; Epley et al., 2004), we expect that readers will initially overestimate the extent to which their privileged information about the speaker’s sarcastic intention is accessible to uninformed addressees. This hypothesis is supported when we find that readers are more likely to attribute their perception of a speaker’s sarcasm onto addressees before (at Time 1) than after feedback (at Time 2). We further expect that both accuracy and narrative feedback will lead to predictions that are more accurate on Time 2 than on Time 1, compared with the condition in which this feedback is absent (control). Finally, we expect that this adjustment (from Time 1 to Time 2) effect is stronger when readers receive accuracy rather than narrative feedback about their performance because we expect that the accuracy feedback will receive more attention than the narrative feedback, partly because the corrective intent and the applicability of the latter type need to be inferred by perceivers themselves. In addition to testing these hypotheses, we anticipate possible individual differences in readers’ propensity to engage in perspective-taking (see Damen et al., 2019). To this end, we will test the extent to which participants’ self-reported propensity to engage in perspective-taking predicts their perspective-taking accuracy. The preregistration of our hypotheses and analyses can be accessed via the Open Science Framework (Damen et al., 2018).

### Method

#### Participants and sample size

In this study, we extended the experimental design of Damen et al. (2020) who found medium to large effect sizes. For a medium effect size, the G*Power calculation (version 3.1.9.2) indicated that we would require a sample size of 22 participants per experimental condition to obtain an alpha error probability of .05 and a power of .95. In our preregistration, we described our intention to recruit at least 30 participants per experimental condition. As Damen et al. (2020) recruited around 50 participants per experimental condition, we chose to approximate this number. After a period of 3 months, we reached a total sample size of 149 undergraduates. Seven participants were excluded either because they recognised the voice-actor (the fifth author) (*N* = 5) or because they were non-native speakers of the language of the experiment (Dutch, *N* = 2). The remaining 142 participants (105 women, 37 men, *M_age_* = 21.57 years, age range=18–38 years) were randomly allocated to the control (*N* = 48), accuracy feedback (*N* = 47), or narrative feedback (*N* = 47) condition.

#### Design

In each condition, participants read 12 stories in which a speaker protagonist (Tom) left a voicemail message on the answering machine of an addressee protagonist. After reading each story, participants listened to the speaker’s actual voicemail and, subsequently, judged addressees’ perception of the speaker’s sarcasm (1 = *definitely as sincere*, 7 = *definitely as sarcastic*), both before (Time 1) and after (Time 2) they received written feedback about their first perspective-judgement. This resulted in a 3 (*Condition*: control, accuracy feedback, narrative feedback) × 2 (*Time*: Time 1, Time 2) mixed design in which *Condition* was treated as a between-subjects factor and *Time* as a within-subjects factor.

#### Procedure and materials

The Dutch materials of this experiment are accessible via osf.io/kpw6u. Participants were invited to the lab and were asked to sit in soundproof cubicles. All participants gave their consent before participating in the experiment. Participants read 12 stories (and a practice item) on a computer screen that were presented in Qualtrics^XM^. This software was also used to collect participants’ responses. All stories described an event in the life of Tom. For instance, in the story “The Dance Class,” participants read the following (English translations of Dutch originals):

Tom was on his way to the first night of his ballroom dancing class when he saw Eileen, an old friend from his dorm last year. When he told her that he was on his way to a ballroom dancing class, she excitedly replied, “I’m thinking of taking that class, but I can’t make it to tonight’s class—I am having dinner with friends. Could you call me when you get back and tell me how it is?”

Subsequently, participants learned that Tom’s experience had been either negative (e.g., “[. . .] the instructor spent the entire time taking attendance and filling out lengthy forms and questionnaires.”) or positive (e.g., “[. . .] the instructor spent the entire time teaching the class fun, new dances.”). Both experiences followed with Tom leaving a voicemail on the answering machine of his friend. In “The Dance Class” story, Tom left the following voicemail message:

“Eileen, this is Tom. Hope you enjoyed your dinner. About that ballroom dancing class: Judging from tonight’s class, I can’t think of better ways to spend my Tuesday evenings. Anyways, give me back a call and I’ll fill you in on the details. Bye.”

The voicemail messages were presented to participants auditorily. After reading the speaker’s experience, participants navigated to a blank page on which the speaker’s voicemail was played over headphones. We re-used the 12 voicemails from Damen et al. (2020) who demonstrated the validity of the voicemails.

Immediately after listening to Tom’s voicemail, participants navigated to a new screen on which they indicated how the addressee protagonist (Tom’s friend) would perceive the voicemail message they just heard (1 = *definitely as sincere*, 7 = *definitely as sarcastic*; middle points on the scale did not contain labels). This constituted the first measurement of participants’ judgement of the addressee’s perception of sarcasm (Time 1). Stories were grouped together in digital booklets that were presented in Qualtrics^XM^, with half the stories describing a positive event, whereas the other half described a negative event. We created four versions of these digital booklets: The first digital booklet contained a random order of negative versus positive events (Booklet 1), and another one contained its mirror image (Booklet 2). In addition, for each booklet, we created a version that contained a reversed order of the events. We were interested in the extent to which readers accurately learned to engage in perspective-taking. Therefore, in contrast to Damen et al. (2020), we only focused on those instances in which readers’ egocentric perspective *diverged* from the addressee protagonists. This was only the case for the stories in which readers’ privileged information suggested that Tom was being sarcastic (negative events). In these instances, readers could engage in egocentric projection by wrongly assuming that their perception of Tom’s sarcasm was shared by the uninformed addressee protagonists. As readers’ interpretation of Tom’s message corresponded to addressees’ perspective when readers’ privileged information suggested Tom was being sincere (positive events), we treated these trials as fillers.

#### Feedback manipulation

**Figure 1.**
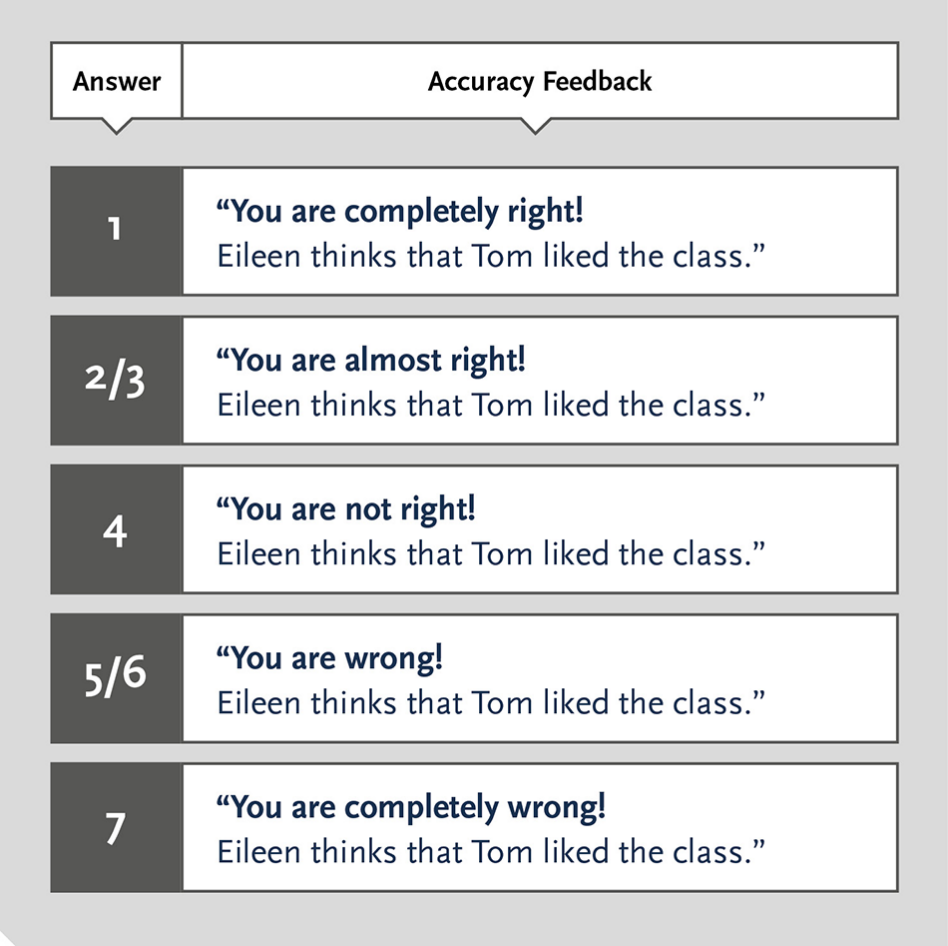
Example of the accuracy feedback participants received for the scenario “The Dance Class.” The type of feedback depended on participants’ choice on the 7-point scale (1 = *definitely as sincere*, 7 = *definitely as sarcastic*).

We manipulated which feedback (accuracy, narrative, none) participants received after their first judgement about the addressee’s perception of sarcasm. Participants received the feedback in Qualtrics^XM^. All feedback was presented visually and, hence, non-auditorily, to participants. In the accuracy feedback condition, participants only received information about the accuracy of their judgement. This feedback was tailor-made in the sense that participants were informed about the extent to which their judgement was inaccurate. For all stories, addressee protagonists had no reason to interpret the speaker’s message as sarcastic. This means that only those readers who predicted that the addressees would interpret the message definitely as sincere (1) made an accurate perspective-taking attempt. Therefore, the accuracy feedback ranged from “You are completely right!” (1) to “You are completely wrong!” (7), depending on participants’ answer on the 7-point scale (1 = *definitely as sincere*, 7 = *definitely as sarcastic*). For example, those who selected “2” on the 7-point scale were informed that their answer was almost accurate. An example of the accuracy feedback for “The Dance Class” story is presented in Figure 1. By tailoring the accuracy feedback participants’ predictions, we aimed to stimulate participants’ adjustment away from the self-perspective by reducing their insecurity about the ambiguousness of the voicemail.

In contrast, participants in the narrative feedback condition only received narrative feedback about the accuracy of their perspective-judgement, regardless of their choice on the 7-point scale. This feedback constituted a follow-up text that described the addressee’s sincere interpretation of Tom’s voicemail, advancing the development of the story. For instance, in “The Dance Class” story, participants could derive from Eileen’s behavioural response to Tom’s voicemail that she thought that Tom had enjoyed attending the class:

After saying goodbye to her friends, Eileen cycled home. She decided she was going to search for her dancing shoes the minute she would arrive at home. She could hardly wait to join Tom in the dance class. If Tom liked the dance class, she definitely would like it too.

Participants in the control condition did not receive feedback about their first assessment of addressees’ perception of sarcasm. Subsequently to their first judgement, these participants read a follow-up text that described the addressee’s thoughts and actions that did not target her interpretation of the voic email:

After saying goodbye to her friends, Eileen cycled home. She and her friends enjoyed dinner. They had known each other since high school and had built up a close friendship. Although they only saw each other a few times a year, it was always as if they never had been apart.

To examine the uptake of the feedback, participants in all three conditions subsequently re-judged the addressee’s interpretation of the voicemail (1 = *definitely as sincere*, 7 = *definitely as sarcastic*). After this second assessment, we measured the extent to which participants were seriously engaged in the task by asking participants to answer a comprehension question about the story they just read (e.g., “What was the story about?”) with three answer options (e.g., “A. Break dance lesson, B. Street dance lesson, C. Ballroom dance lesson). We made sure that all 12 comprehension questions did not target participants’ privileged information and that the level of difficulty varied across all questions. Participants who answered the comprehension question incorrectly were informed to read the stories and to listen to the voicemails more carefully. Participants answered on average 11 out of 12 questions correctly (*M* = 10.52, *SD* = 1.07). We examined the extent to which participants’ accuracy scores on the comprehension questions differed between the experimental conditions. As the accuracy scores were non-normally distributed in all three conditions,^2^ we performed a non-parametric Kruskal–Wallis test to investigate this relationship. This test showed that the number of correct responses differed between conditions, *H*(3) = 9.73, *p* < .01. Pairwise comparisons with adjusted *p*-values indicated that participants answered more comprehension questions correctly in the narrative feedback condition (*M* = 10.81, *SD* = 0.95) than in the accuracy feedback condition (*M* = 10.13, *SD* = 1.15) (*p* < .01). The accuracy scores did not differ between the control (*M* = 10.63, *SD* = 1.00) and the two feedback conditions (*p* > .05). Arguably, this difference is not so surprising, given the nature of the different feedback types. It can be argued that readers in the narrative feedback condition spent more time processing the information because they had to infer the mismatch in perspectives from a description of addressees’ true perspective (see also Weingartner & Klin, 2005). This in contrast to the readers who were not (control) or were explicitly informed (accuracy feedback) about the inaccuracy of their judgement and, thereby, could have spent less time on the information presented in the stories.

#### Participants’ perspective-taking tendency

As a final step in the experimental procedure, we asked participants to fill out a questionnaire measuring the extent to which participants themselves thought they had acknowledged addressees’ perspective. The questionnaire contained eight items (e.g., “I was aware that Tom’s friends could interpret the voicemail messages differently from me”) that were alternated by seven filler questions (e.g., “I liked to read the stories”) and measured on a 7-point scale (1 = *totally disagree*, 7 = *totally agree*). The individual items are presented in Table 1. The scale had a moderate reliability (Cronbach’s α = .64). Factor analysis showed that the scale could not be divided into meaningful subsets or into a subset that improved the reliability of the scale. Hence, we treated it as a single scale and report below on the average score of the eight items. After filling out the questionnaire, we collected participants’ demographics, debriefed participants about the purpose of the experiment, and thanked them for their participation.

**Table 1.** Items of participants’ perspective-taking tendency scale.

While reading the stories, listening to the voicemails, and answering the questions that followed the voicemails: 1. I especially took into account what I knew about Tom’s experience (R)
2. I found it difficult to imagine how Tom’s friends would interpret the voicemails (R)
3. I especially took into account what Tom’s friends knew about Tom’s experience
4. I could easily imagine how Tom’s friends would interpret the voicemails
5. I was especially aware of what Tom’s friends knew about Tom’s experience
6. I tried to imagine as much as possible how Tom’s friends would understand the voicemails
7. I was especially aware of what I knew about Tom’s experience (R)
8. I was aware that Tom’s friends could interpret the voicemail messages differently from me

*Note*. Items (R) were recoded before analysis.

### Results

#### Feedback and perspective adjustments

The anonymized dataset of this experiment is accessible via osf.io/kpw6u. In our preregistration, we planned to perform an analysis of variance (ANOVA) and a subsequent linear mixed effects analysis (LMER) to control for random item and subject effects. We also performed exploratory analyses to examine learning effects and the relationship between participants’ self-reported perspective-taking tendency and their actual perspective-taking behaviour. Below, we report on the ANOVA and we provide a summary of the learning effects findings. Detailed information about the statistical procedures and findings of the LMER and exploratory analyses can be found in the Supplementary Material.

We computed a mean perceived sarcasm score of participants’ first (Time 1) and second (Time 2) judgement of addressees’ perception of the speaker’s sarcasm. Exploratory analyses that included the difference score between the two mean ratings revealed three outliers in the control condition and one outlier in the accuracy feedback condition (the deviance ranged from −4.78 to 2.99). When these outliers were excluded, normality improved and the data in the accuracy feedback (*Z_skewness_* = –0.07, *Z_kurtosis_* = –0.89), narrative feedback (*Z_skewness_* = 0.94, *Z_kurtosis_* = –0.70), and control condition (*Z_skewness_* = 0.96, *Z_kurtosis_* = –1.02) were normally distributed. To adhere to our preregistration, we will report the findings of our analyses that did not include these three outliers. To examine whether the outliers affected our findings, we re-ran our analyses and report whether the findings are different when the outliers were included.

**Figure 2.**
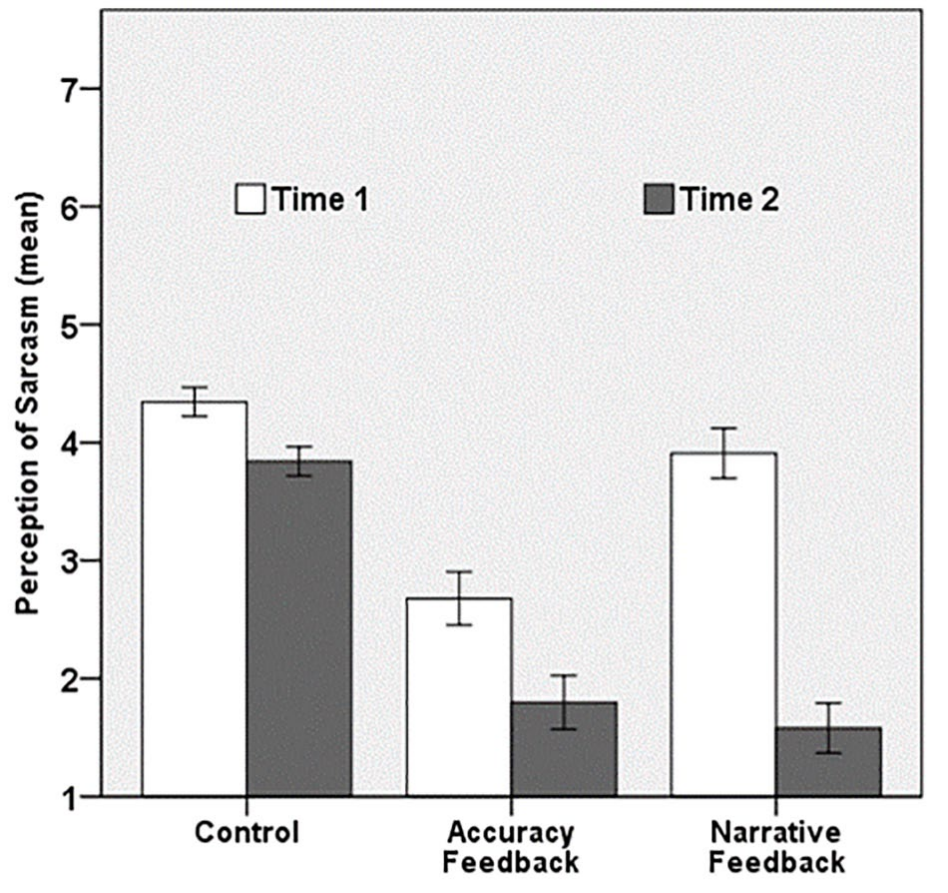
Mean scores of participants’ judgement of addressees’ perception of sarcasm (1 = *definitely as sincere*, 7 = *definitely as sarcastic*) as a function of *Time* (Time 1, Time 2) and *Condition* (control, accuracy feedback, narrative feedback).

We submitted the two mean scores (Time 1, Time 2) to a mixed ANOVA in which *Condition* (control, accuracy feedback, narrative feedback) was treated as a between-subjects factor and participants’ judgement of addressees’ perception of sarcasm (*Time*; Time 1, Time 2) as a within-subjects factor. The means of participants’ judgement of addressees’ perception of sarcasm as a function of *Time* and *Condition* are presented in Figure 2.

In line with our first hypothesis, participants thought addressees would perceive the speaker’s sarcasm more at their first (*M_Time_*_1_ = 3.64, *SD* = 1.17) than at their second perspective-judgement (*M_Time2_* = 2.40, *SD* = 1.27), *F*(1, 134) = 249.48, *p* < .001, $\eta_{p}^{2}$=.65. Furthermore, participants’ overall perspective-taking accuracy differed as a function of *Condition, F*(2, 134) = 81.21, *p* < .001, $\eta_{p}^{2}$=.95. Because this main effect included Time 1, which lies before the manipulation, differences between the experimental conditions are irrelevant. It thus makes more sense to look at the interaction effect between the type of feedback (*Condition*) and the time point (*Time*). We predicted no difference between participants’ first and second judgement in the control condition, but an improvement between participants’ first and second judgement in the feedback conditions. Results indeed showed that the main effect of *Time* was qualified by a significant interaction with *Condition, F*(2, 134) = 50.49, *p* < .001, $\eta_{p}^{2}$=43. Bonferroni-corrected pairwise comparisons that compared participants’ perspective-taking accuracy of their second perspective-judgement showed that participants adjusted their first prediction into a more accurate second prediction after they had received both accuracy (*M_Time2_* = 1.80, *SD* = 0.85, *p* < .001, 95% CI = [–2.43, –1.66]) and narrative feedback (*M_Time2_* = 1.58, *SD* = 0.68, *p* < .001, 95% CI = [–2.65, –1.88], compared with the control condition (*M_Time2_* = 3.84, *SD* = 0.74). In contrast to our predictions, the accuracy of participants’ second prediction did not differ between the two feedback conditions (*p* = .515, 95% CI = [–0.17, 0.60]).

Interestingly, results also showed that participants’ perspective-taking accuracy of their first prediction differed as a function of *Condition*. Pairwise comparisons revealed that participants in the accuracy feedback condition (*M* = 2.68, *SD* = 1.02) thought addressees would perceive less sarcasm at Time 1 than the participants in the narrative feedback (*M* = 3.91, *SD* = 1.00) and control (*M* = 4.34, *SD* = 0.77) conditions (both *p* < .001). At Time 1, the perceived sarcasm scores did not differ between the narrative feedback and control condition (*p* = .084). This may seem strange, but keep in mind that these scores are mean scores over 12 trials. So, participants already received feedback on a previous trial for most trials. All findings remained unchanged when outliers were included in the analysis.

#### Curse of knowledge effect and feedback

We specified in our preregistration that we would explore whether we are able to replicate perceivers’ curse of knowledge effect that has been documented in Damen et al. (2020) and Epley et al. (2004).^3^ This concerns the control condition and the difference between the experimental trials (in which participants have privileged information) and the filler trials. If there is a curse of knowledge effect, the extent to which readers think the addressee perceives sarcasm should be higher for the experimental trials than for the filler trials.

In addition to testing this preregistered assumption, we explored the extent to which feedback reduces this curse of knowledge effect on perspective-taking. To test this, we also compared the sarcasm scores on the experimental trials and the filler trials in the two feedback conditions. As it is most informative to analyse whether participants’ curse of knowledge persists after feedback, we did this for the Time 2 estimations only.

After excluding two outliers in the accuracy feedback condition (deviance 1.98 and 2.15) and seven outliers in the narrative feedback condition (deviance ranged from −0.93 to 1.90), the difference score between the experimental and filler trials at Time 2 was normally distributed in the control (*Z_skewness_* = –0.08, *Z_kurtosis_* = –0.34), accuracy feedback (*Z_skewness_* = 1.60, *Z_kurtosis_* = –0.22), and narrative feedback conditions (*Z_skewness_* = 0.63, *Z_kurtosis_* = 0.36).

**Figure 3.**
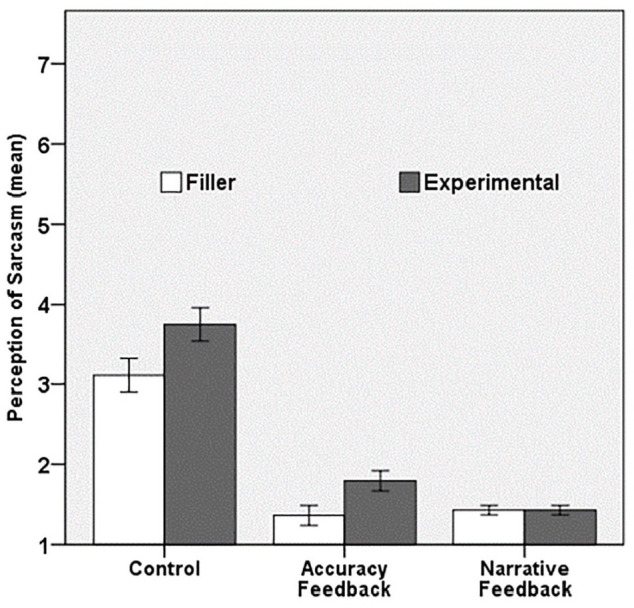
Mean scores of participants’ estimation of addressees’ perception of sarcasm (1 = *definitely as sincere*, 7 = *definitely as sarcastic*) at Time 2 as a function of *Trial* (filler, experimental) and *Condition* (control, accuracy feedback, narrative feedback).

We submitted the two mean scores to a mixed analysis of variance in which *Condition* (control, accuracy feedback, narrative feedback) was treated as a between-subjects factor and participants’ judgement of addressees’ perception of sarcasm at Time 2 (*Trial*: experimental, filler) as a within-subjects factor. The analysis revealed a significant main effect of *Trial, F*(1, 130) = 31.71, *p* < .001, $\eta_{p}^{2}=.20$. At Time 2, participants still thought addressees would perceive the speaker’s sarcasm more when their privileged information suggested the speaker was being sarcastic (experimental; *M* = 2.39, *SD* = 1.29) than when their privileged information suggested the speaker was being sincere (filler; *M* = 2.02, *SD* = 1.06). The analysis further revealed a significant main effect of *Condition, F*(2, 130) = 144.89, *p* < .001, $\eta_{p}^{2}=.69$. More importantly, the main effect of *Trial* was qualified by a significant interaction with *Condition, F*(1, 130) = 8.65, *p* < .001, *$\eta_{p}^{2}=.12$* (see Figure 3). Simple effects analyses revealed that the effect of *Trial* was significant in the control, *F*(1, 130) = 37.31, *p* < .001, *r* = 0.47, and accuracy feedback conditions, *F*(1, 130) = 15.55, *p* < .001, *r* = 0.33, but not in the narrative feedback condition, *F*(1, 130) = 0.00, *p* = 1.00). These findings suggest that—in both the control and accuracy feedback conditions—participants’ privileged information still cursed their ability to estimate a less informed perspective at Time 2. Participants in these two conditions were still more likely to attribute their perception of sarcasm onto addressees when their privileged information suggested the speaker was being sarcastic (experimental trials; *M_control_* = 3.75, *SD* = 0.80; *M_accuracy feedback_* = 1.79, *SD* = 0.97) than when their privileged information suggested the speaker was being sincere (filler trials; *M_control_* = 3.11, *SD* = 0.85; *M_accuracy feedback_* = 1.37, *SD* = 0.61). In contrast, participants receiving narrative feedback were not cursed by their privileged information when they estimated addressees’ perspective at Time 2. These participants thought addressees would perceive the speaker’s voicemail to the same degree for both filler (*M* = 1.43, *SD* = 0.44) and experimental trials (*M* = 1.43, *SD* = 0.40). All findings remained unchanged when the outliers were included in the analyses and when we controlled for the order in which the scenarios (items) were presented to participants.

#### Exploratory analyses

##### Learning effects

When analysing the accuracy of participants’ final judgement of addressees’ perspective (for the final story), we noticed that these judgements also differed as a function of *Condition*, Brown-Forsythe *F*(2, 95.56) = 36.01, *p* < .001. Participants’ final judgement in the accuracy feedback (*M* = 1.46, *SD* = 1.21) and narrative (*M* = 1.38, *SD* = 0.49) feedback conditions was more accurate than participants’ final judgement in the control condition (*M* = 3.18, *SD* = 1.48), *t*(60.54) = –7.30, *p* < .001. In addition, the accuracy of participants’ final judgement did not differ between the accuracy and narrative condition, *t*(59.29) = –0.38, *p* = .703.

### Discussion

Experiment 1 showed that perceivers improved the accuracy of their social predictions when they received feedback about their perspective-taking performance. In line with our expectations, perceivers initially overestimated the extent to which their perception of a speaker’s sarcastic intention was accessible to uninformed addressees. Hence, the results of Experiment 1 replicated perceivers’ curse of knowledge effect (Damen et al., 2020; see also Epley et al., 2004; Keysar, 1994; Weingartner & Klin, 2005, 2009). Perceivers were more likely to assume that an uninformed addressee would perceive a speaker’s sarcasm when perceivers’ privileged information suggested that the speaker was being sarcastic rather than being sincere. Interestingly, our findings revealed that this egocentricity bias was eliminated after narrative feedback. This shows that narrative feedback can lift the curse of knowledge effect. Accuracy feedback did not have the same effect, because even though it improved the accuracy, there was still a curse of knowledge effect in that condition.

Although perceivers in the control condition did not receive feedback about the accuracy of their predictions, our findings indicated that they also adjusted their first prediction to a more accurate second prediction of addressees’ perspective. This adjustment could have been the result of perceivers reflecting on their earlier assessment and subsequently coming to a more accurate conclusion (although these adjustments were still less accurate compared with the adjustments made by the perceivers who received feedback). In Experiment 2, we ask perceivers to judge addressees’ perspective only once for each story. Thereby, we examine whether participants’ improved accuracy at Time 2 constituted a true transfer of learning and did not arise due to participants benefiting from having to re-think about their first assessment.

## Experiment 2

The aim of Experiment 2 is twofold. First, we examine whether the perspective-adjustments in the feedback conditions reported in Experiment 1 were due to the experimental manipulation and not due to participants judging addressees’ perception twice for each experimental item. To investigate this, we allocated participants to two additional experimental conditions in which participants judged addressees’ perspectives only once (at Time 2) for each scenario. In particular, we allocated participants either to a one-shot control or to a one-shot narrative feedback condition. These additional experimental conditions were exact replications of the control and narrative feedback conditions reported in Experiment 1, except for the fact that participants now judged addressees’ interpretation of the voicemail only once for each scenario (item). We hypothesise that participants’ judgements of addressees’ perception of sarcasm when participants’ privileged information suggested that the speaker was being sarcastic (negative events) will not differ between the two-shot control condition (Experiment 1) and the one-shot control condition (Experiment 2), nor between the two-shot narrative feedback condition (Experiment 1) and the one-shot narrative feedback condition (Experiment 2).

Furthermore, we aim to replicate the finding that narrative feedback can lift the curse of knowledge effect. We expect that participants will be less likely to misattribute their perception of the speaker’s sarcasm onto addressees when participants receive information about addressees’ (sincere) interpretation of the voicemail (narrative feedback) than when this feedback is absent (control).

### Method

#### Participants and sample size

As in Experiment 1, we aimed to recruit 50 participants per experimental condition. After 3 months of data collection, 93 undergraduates were recruited. Three participants were excluded because they recognised the voice-actor (the fifth author). The remaining participants were randomly allocated to either the one-shot control (*N* = 44) or the one-shot narrative feedback condition (*N* = 46). For the two-shot control (*N* = 48) and two-shot narrative feedback (*N* = 47) conditions, we used the data from Experiment 1. Our analyses are thus based on 185 undergraduates (125 women, 60 men, *M_age_* = 21.57 years, age range=17–32 years). All participants gave their consent before participating in the experiment and received course credits for their participation.

#### Design, materials, and procedure

We replicated the experimental design of Experiment 1. Hence, detailed information about the study’s design, materials, and procedure can be found there. The only difference is that we now measured participants’ judgements of addressees’ perception of the speaker’s sarcasm only once for each experimental item (1 = *definitely as sincere*, 7 = *definitely as sarcastic*). Participants in the one-shot control condition judged addressees’ perception immediately after listening to the speaker’s voicemail (as in Damen et al., 2020; Epley et al., 2004). In contrast, participants in the one-shot narrative feedback condition read an additional text before judging addressees’ perception. This text described addressees’ reaction to the speaker’s message and was an exact replications from the text used in the two-shot narrative feedback condition (Experiment 1). In this way, we were able to investigate whether reading the story developing allowed participants to reflect on their initial judgement (even when no perspectives were targeted in the control text), affecting their perspective-taking accuracy. Participants could infer from addressees’ behavioural response that—for both positive and negative experiences—addressees thought Tom had been sincere about his past experience. We contrasted participants’ judgements of addressees’ perception of sarcasm collected in the one-shot control and one-shot narrative feedback conditions against participants’ sarcasm scores collected in Experiment 1. More specifically, for the two-shot conditions, we re-used the data that were collected on the second time measurement (Time 2) in Experiment 1. Hence, our analyses will be based on a fully between-subjects design in which participants either received information about addressees’ true uptake of the speaker’s message (*Narrative Feedback*: present, absent) or assessed addressees’ perspective either once or twice for each story (*Time Measurement*: one-shot, two-shot).

In both one-shot conditions, each story ended with a comprehension question measuring participants’ attentiveness while reading the stories and listening to the voicemails. Participants in the one-shot control and one-shot narrative feedback conditions answered almost all questions correctly (*M* = 10.61, *SD* = 0.92), and the number of correct responses did not differ between experimental conditions, *H*(1) = 0.21, *p* = .646. Subsequently, participants filled out the perspective-taking tendency scale from Experiment 1 (Cronbach’s α = .63.), noted down their demographics, were debriefed about the purpose of the experiment, and were thanked for their participation.

### Results

#### Feedback and perspective adjustment

The anonymized dataset of this additional study is accessible via osf.io/kpw6u, and our preregistered analyses are accessible via osf.io/vbsyz (Damen et al., 2018). Following the statistical procedures of Experiment 1, we computed a mean sarcasm score of participants’ judgement of addressees’ perception of the speaker’s sarcasm for the scenarios in which participants’ privileged information suggested that the speaker was being sarcastic (negative events; experimental trials). Recall that, for the two-shot control and two-shot narrative feedback conditions, we used participants’ responses that were collected in Experiment 1 at Time 2. Exploratory analyses revealed one outlier (the deviance was 3.25) in the two-shot narrative feedback condition. After excluding this outlier, the data in the two-shot narrative feedback (*Z_skewness_* = 2.01, *Z_kurtosis_* = –0.32) and one-shot narrative feedback (*Z_skewness_* = 3.02, *Z_kurtosis_* = –0.01) conditions were still positively skewed. The data in the one-shot control (*Z_skewness_* = –1.45, *Z_kurtosis_* = 0.65) and two-shot control (*Z_skewness_* = –0.47, *Z_kurtosis_* = –1.29) conditions were normally distributed. As the assumption of normality was violated for the narrative feedback conditions, we re-examined our hypotheses employing a linear mixed effects analysis (see Supplementary Material).

We submitted the average sarcasm score to a univariate ANOVA in which we examined the influence of the factors *Time Measurement* (one shot, two-shot) and *Narrative Feedback* (absent, present) on participants’ judgement of addressees’ perception of sarcasm. The analysis revealed a significant effect of *Narrative Feedback* on participants’ judgement of addressees’ perception of sarcasm, *F*(1, 181) = 382.72, *p* < .001, $\eta_{p}^{2}=.68$. Participants thought addressees would perceive the speaker’s sarcasm more when narrative feedback was absent (*M* = 3.91, *SD* = 0.94) than when it was present (*M* = 1.58, *SD* = .67). The main effect of *Time Measurement, F*(1, 181) = 2.06, *p* = .153, and the interaction between *Time Measurement* and *Narrative Feedback, F*(1, 181) = 1.93, *p* = .166, were both non-significant. The perceived sarcasm scores did not differ between the one-shot (*M* = 2.81, *SD* = 1.53) and two-shot (*M* = 2.68, *SD* = 1.32) conditions, and this difference remained the same whether participants read addressees’ uptake of the speaker’s message or not. All findings remained unchanged when the outlier was included in the analysis.

#### Curse of knowledge effect and feedback

We examined whether we could replicate participants’ curse of knowledge effect from Experiment 1. We hypothesised that participants would project their perception of a speaker’s sarcasm onto addressees more when their privileged information suggested the speaker was being sarcastic (negative events; experimental trials) than when their privileged information suggested the speaker was being sincere (positive events; filler trials). We further expected that this effect would be qualified by our feedback manipulation. In particular, we expected that the difference in participants’ attribution of a speaker’s sarcasm for positive and negative events would remain in the one-shot condition in which participants did not receive insight into addressees’ interpretation of the speaker’s message, but would disappear when participants did receive addressees’ uptake of the message. To examine this hypothesis, we computed a mean perceived sarcasm score for participants’ estimation of addressees’ perception of the speaker’s sarcasm for both negative and positive events. Exploratory analyses that included the difference score of negative and positive events revealed 13 outliers on the one-shot condition with narrative feedback (deviance ranged from −0.59 to 1.41) and one outlier in the one-shot condition without narrative feedback (deviance = –4.02). In compliance with our preregistration and Experiment 1, we excluded outliers to improve the normal distribution of the data. After excluding the outliers, the data were normally distributed in the one-shot (*Z_skewness_* = –0.91, *Z_kurtosis_* = 0.46) and two-shot control conditions (*Z_skewness_* = –0.08, *Z_kurtosis_* = –0.34), but were still positively skewed and heavily tailed in the one-shot narrative feedback condition (*Z_skewness_* = 9.26, *Z_kurtosis_* = 22.80) and in the two-shot narrative feedback condition (*Z_skewness_* = 4.03, *Z_kurtosis_* = 5.26). We submitted the two mean scores to a mixed analysis of variance in which *Narrative Feedback* (absent, present) was treated as a between-subjects factor, and participants’ judgement of addressees’ perception of sarcasm (*Trial*: experimental, filler) as a within-subjects factor. Results revealed a main effect of *Trial, F*(1, 75) = 14.96, *p* < .001, *ƞ_p_*^2^ = 0.17, that was qualified by a significant interaction with *Narrative Feedback, F*(1, 75) = 10.96, *p* = .001, *ƞ_p_*^2^ = 0.13. Participants thought addressees would perceive the speaker’s sarcasm more on experimental trials (negative events) where their privileged information suggested the speaker was being sarcastic (*M* = 2.95, *SD* = 1.59) than on filler trials (positive events) where their privileged information suggested the speaker was being sincere (*M* = 2.39, *SD* = 1.17). Confirming our hypothesis, the simple effect analysis revealed that this difference remained significant when participants did not receive narrative feedback, *F*(1, 169) = 61.02, *p* < .001, $\eta_{p}^{2}=.27$, but that this difference disappeared when participants did receive insight into addressees’ true uptake of the speaker’s message, *F*(1, 169) = 0.64, *p* = .425. All findings remained unchanged when the outliers were included in the analyses and when we controlled for the order in which the items (scenarios) appeared to participants.

Additional analyses were performed to control for random item and subject effects and to examine the relationship between participants’ self-reported perspective-taking tendency and their actual perspective-taking behaviour. Detailed information about the statistical procedures and results of our exploratory analyses can be found in Supplementary Material.

## General discussion

This study examined the extent to which performance feedback stimulates perceivers to make accurate social predictions. In particular, we set out to investigate whether and how feedback affects perceivers’ ability to take the perspective of a person who is less informed than they are. In addition, we examined the extent to which different feedback types (accuracy or narrative) affect perceivers’ perspective-taking performance. We investigated this question by replicating and extending the experimental design of Damen et al. (2020) in which perceivers judged addressees’ perception of a speaker’s message. Findings indicated that perceivers learned from the feedback they received. After feedback, perceivers made predictions that were more accurate, meaning that they were less likely to assume that addressees would also perceive the speaker’s sarcasm.

Experiment 2 further evidenced the effectiveness of feedback to improve perceivers’ social judgement by ruling out the possibility that improvements in perspective-taking were due to task demands. In Experiment 2, perceivers judged addressees’ perspective only once after they did (one-shot narrative feedback) or did not (one-shot control) receive insight into addressees’ uptake of the speaker’s message. Perceivers receiving narrative feedback made more accurate predictions about addressees’ perspective than the perceivers without this feedback did.

This study also examined whether the type of performance feedback (accuracy, narrative) affects the extent to which perceivers improve the accuracy of their predictions. We reasoned that the corrective intent of narrative feedback might be less clear to perceivers because they need to infer counterfactual information from a description of addressees’ true perspective (e.g., Chandler, 2003; Ellis, 2009; Nicholas et al., 2001; Van Beuningen et al., 2008). We therefore assumed that perceivers’ adjustments made to the self-perspective would be less accurate after narrative rather than accuracy feedback. In contrast to this expectation, however, perceivers’ adjustments did not depend on the type of feedback they received. More specifically, perceivers who were informed about the extent to which their first prediction was inaccurate (accuracy feedback) were just as accurate the second time around as the perceivers who had to infer their perspective-taking accuracy from a description of addressees’ true perspective (narrative feedback).

We further showed that accuracy feedback, but not narrative feedback, increased accuracy on the first prediction of later trials (see Supplementary Material). This means that people improved their accuracy over time. It could be argued that these results were caused by perceivers learning to uphold a different strategy to infer the addressee protagonists’ perspective based on the type of feedback they received. Recall that perceivers in the accuracy feedback condition received tailor-made feedback about the inaccuracy of their judgement based on their answer on the 7-point scale. Hence, these perceivers were explicitly informed about the extent to which their egocentric anchoring was inaccurate. Perceivers in the accuracy feedback condition made better perspective-taking deductions on first trials, decreasing the overall level of egocentrism (over time). In contrast, perceivers in the narrative feedback condition had to deduce the incorrectness of their judgement from a description of addressees’ behavioural response to the messages. Perceivers in the narrative feedback condition, therefore, could have been more cautious to assume addressees’ sincere interpretation until they had actually inferred addressees’ uptake of the messages. Given the ambiguity of the judgements, it is reasonable to assume that the readers in the narrative feedback condition kept assessing the target’s feelings based on an (incorrect) self-anchor. Especially in ambiguous situations, readers are expected to judge other people’s perspectives using their own knowledge as a frame of reference (e.g., Epley, Morewedge, & Keysar, 2004; Krueger, 2003; see also “extratarget strategies” such as egocentric projection and stereotyping in Ames, 2005). This reliance on self-knowledge is argued to decrease in light of behavioural counterevidence (for a review, see Ames, 2005). Our findings from the narrative feedback condition confirm this suggested pattern. Perceivers receiving narrative feedback only adjusted their egocentric anchor for the second measurement of that same scenario, but they did not transfer this adjustment to later scenarios. Examining which perspective-taking strategy readers upheld to infer addressees’ perspective is beyond the scope of this research. Future research might examine this question using more qualitative methods to capture readers’ perspective-taking strategy at each step during the task.

An important question that arises here is whether the increase in accuracy on participants’ first responses in the accuracy feedback condition constitutes a learning effect. As we have previously seen, the accuracy scores of participants’ second judgements did not differ between the two feedback conditions, nor did the accuracy of their final judgement (see Supplementary Material for more detail). More importantly, we showed that, regardless of this increase in accuracy on first responses, participants in the accuracy feedback condition still misattributed their perception of the speaker’s sarcasm the second time around, whereas participants in the narrative feedback condition did not (a point we return to, shortly). These findings seem to suggest that accuracy feedback increases participants’ accuracy because they learn how the task should be performed. In this sense, accuracy feedback does not seem to suffice to reduce participants’ egocentric projection and, as a result, their interpersonal accuracy.

### Curse of knowledge effect and feedback

This study replicated the curse of knowledge effect on perspective-taking (e.g., Damen et al., 2020; Epley et al., 2004; Keysar, 1994; Weingartner & Klin, 2005, 2009). Perceivers were more likely to attribute their perception of the speaker’s sarcasm onto addressees when their privileged information suggested the speaker was being sarcastic than when their privileged information suggested the speaker was being sincere. More importantly, we further showed that this curse of knowledge effect was eliminated when perceivers received insight into another person’s mental state. That is, when perceivers experienced addressees’ uptake of the speaker’s message, they were less likely to use their privileged knowledge to judge the perspective of the addressees. In this way, our findings extend those by Weingartner and Klin (2005), who found that perceivers took more time to process addressees’ perspective because it presented them with counterfactual information they needed to reconcile. We have shown that this online processing of counterfactual information translated to perceivers explicitly acknowledging addressees’ perspective when judging their viewpoint. Important to note here is that perceivers’ bias was only eliminated after they experienced addressees’ uptake of the speaker’s message (narrative feedback), but not after they received accuracy feedback about their performance. Hence, even though *perspective-taking accuracy* improved for both feedback types, perceivers’ *bias* was not eliminated after accuracy feedback (see also discussion in Eyal et al., 2018). These findings correspond to previous studies by Camerer et al. (1989), Thompson and DeHarpport (1994), and Eyal et al. (2018), who showed that receiving or *getting* (Eyal et al., 2018) information that allows perceivers to make accurate inferences can reduce—or in this study eliminate—perceivers’ egocentric bias during social judgement. In this way, this study provides promising directions for future studies aimed to eliminate related cognitive biases (see Pronin, 2006, for a review).

Following from this line of argument, we question whether feedback helps perceivers to learn more about their overall perspective-taking proficiency. Findings from perceivers’ self-reported perspective-taking tendency suggest that perceivers receiving feedback after their first prediction did not feel that they took addressees’ perspective more into account than the participants who did not receive this feedback. In contrast, participants in the one-shot narrative feedback condition, who judged addressees’ perspective (explicitly) *after* receiving their uptake of the ambiguous messages, reported a higher perspective-taking tendency than participants in other conditions. Furthermore, we saw that self-reported measures did not predict actual perspective-taking behaviour in Experiment 1, but they did in Experiment 2. We believe that it is not surprising that participants in the two-shot feedback condition felt they were less proficient in taking perspectives into account than participants in the one-shot feedback and control conditions. After all, the former group of participants *did* receive information about the proficiency of their perspective-taking performance. Therefore, participants in the one-shot narrative feedback condition might not have been aware of any corrections they had made to the self-perspective (after hearing the speaker’s voicemail) to represent addressees’ interpretation of the message (after reading addressees’ uptake). Of course, whether these participants went through such a correction phase would only have been apparent if we had tracked their online perspective-taking behaviour (e.g., Weingartner & Klin, 2005, 2009) because we only measured their explicit judgement *after* they read addressees’ interpretation. A tentative explanation we draw here is that the feedback provided in the two-shot conditions might have lowered participants’ confidence in their perspective-taking proficiency (e.g., see Ryback, 1967), in contrast to the participants in the one-shot narrative condition and control condition. Perceivers’ self-reported perspective-taking tendency in Experiment 2 seems to support this tentative explanation. Although perceivers experiencing addressees’ uptake of the message (narrative feedback conditions) were equally accurate in their predictions of addressees’ perspective, those who received this information after their first prediction (two-shot narrative feedback) reported a lower perspective-taking tendency than the perceivers who received insight into addressees’ perspective before they made any prediction at all (one-shot narrative feedback condition). This reduction in confidence can be especially beneficial in terms of increasing readers’ perspective-taking accuracy. Perceivers’ overconfidence in their ability to predict another person’s mental state often stands in the way of accurate predictions (Eyal et al., 2018; Savitsky et al., 2011; Swann & Gill, 1997). Future research might further examine the relation between feedback and perceivers’ confidence, and how this confidence is related to perspective-taking accuracy.

### Egocentric anchoring and adjustment

The findings of this research are in line with the egocentric anchoring and adjustment process of perspective-taking. In this study, perceivers who received information about another person’s true perspective were more likely to *adjust* away from an egocentric interpretation than the perceivers who did not receive this insight. Interestingly, even though explicit accuracy feedback about performance improved their perspective-taking, our findings showed that these adjustments were still “insufficient” (e.g., Barr & Keysar, 2005; Epley, 2008; Epley & Gilovich, 2004, 2006; Epley et al., 2004; Jacowitz & Kahneman, 1995; Quattrone, 1982; Tversky & Kahneman, 1974). That is, perceivers receiving accuracy feedback were still biased by their own perception of the speaker’s message. Only perceivers receiving narrative feedback about their performance were able to inhibit their own uptake of the message to appreciate the less informed perspective of addressees. We encourage future research to examine whether this performance feedback helps perceivers to improve the accuracy of their social predictions over time and whether this improvement will transfer to other perspective-taking activities by helping perceivers to select a more accurate approach to infer another person’s viewpoint.

## Supplemental Material

sj-docx-1-qjp-10.1177_1747021820987080 – Supplemental material for Lifting the curse of knowing: How feedback improves perspective-taking Click here for additional data file. Supplemental material, sj-docx-1-qjp-10.1177_1747021820987080 for Lifting the curse of knowing: How feedback improves perspective-taking by Debby Damen, Marije van Amelsvoort, Per van der Wijst, Monique Pollmann and Emiel Krahmer in Quarterly Journal of Experimental Psychology

## Notes

1. Details of the perspective-focus instructions are accessible via osf.io/kv5mu.

2. Accuracy feedback (*Z_skewness_* = 0.02; *Z_kurtosis_* = –1.35), narrative feedback (*Z_skewness_* = –2.07; *Z_kurtosis_* = 0.77), and control conditions (*Z_skewness_* = –2.20, *Z_kurtosis_* = 0.70).

3. In our preregistration, we specified that we would test participants’ curse of knowledge effect on perspective-taking in our one-shot control condition (see Experiment 2). However, with regard to its integrity, we chose to analyse this effect also in Experiment 1.
