# Supplementary material for: Lifting the curse of knowing: How feedback improves perspective-taking
Source: Q J Exp Psychol (Hove). 2021 Feb 4;74(6):1054–69. doi: 10.1177/1747021820987080 (PMC8107504; doi:10.1177/1747021820987080)
Supplement: sj-docx-1-qjp-10.1177_1747021820987080 – Supplemental material for Lifting the curse of knowing: How feedback improves perspective-taking [file sj-docx-1-qjp-10.1177_1747021820987080.docx]

**Supplementary Materials**

Lifting the Curse of Knowing: How Feedback Improves Readers’ Perspective-Taking

Damen, Van Amelsvoort, Van der Wijst, Pollmann, Krahmer

**Experiment 1**

**Linear Mixed Effects Analyses**

**Feedback and Perspective Adjustments.** In addition to our mixed analysis of variance, we preregistered that we would perform linear mixed effects analyses to control for random item (scenarios) and subject effects (e.g., Baayen, Davidson, & Bates, 2008; Baayen & Milin, 2010). For this analysis, we used the LMER function from the lme4 package in R (version 3.5.1, CRAN project; R Core Team, 2017). We construed four models to obtain the comparisons appertaining to our hypotheses and used a Bonferroni correction (α ≤ .013) to correct for multiple comparisons. We started the analyses by construing the models with a maximal random effects structure (Barr, Levy, Scheepers, & Tily, 2013). These maximal models included *Condition* (control, accuracy feedback, narrative feedback), *Time* (Time 1, Time 2), and *Condition***Time* interaction as fixed factors, and random intercepts and slopes for both subjects and items. When the model did not converge, we excluded random slopes with the lowest variance until the model did converge (Barr et al., 2013). For all models, only the model containing random intercepts for both subjects and items reached convergence. For these intercept-only models, we estimated the confidence intervals and *p*-values by parametric bootstrapping over 100 iterations (Bates, Mächler, Bolker, & Walker, 2015). The estimated coefficients, standard errors and the structure of the models are presented in Table 1.

The linear mixed effect analyses replicated the results from our mixed analysis of variance. All three conditions showed a main effect of *Time*, indicating that readers’ second prediction of the addressee protagonist’s perspective was more accurate than their first. In addition, the accuracy of these second predictions differed as a function of *Condition*. Readers who received either accuracy or narrative feedback about the accuracy of their first prediction were more accurate the second time around than readers who did not receive such feedback (control). Moreover, the accuracy of readers’ second prediction did significantly differ between the feedback conditions and the control, but not between the two feedback conditions themselves. Furthermore, readers who received accuracy feedback made more accurate first predictions than readers in both the narrative feedback and control condition. The linear mixed effect analysis showed, however, a slightly different pattern when examining the influence of *Condition* on the degree to which readers adjusted their perspective. Whereas the mixed analysis of variance showed that readers in the control condition adjusted their perspective less than the readers in both feedback conditions, the linear mixed effects analyses revealed that the degree to which readers adjusted their perspective only *tended* to differ between the control and accuracy feedback condition (i.e., this difference reached significance at the 95% CI). Remaining findings of the ANOVA were also reflected in the LMER analyses: the perspective-adjustments significantly differed between the narrative feedback condition and the control, and between the narrative feedback condition and the accuracy feedback condition.

**Curse of Knowledge Effect**. For these linear mixed effect analyses, we followed the same procedure as described above. We construed three models to examine whether readers significantly attribute more sarcasm to addressees at Time 2 when their privileged information suggests the speaker is being sarcastic (experimental trials) than when their privileged information suggests that the speaker is being sincere (filler trials), and whether this attribution of sarcasm differs as a function of *Condition*. We used a Bonferroni correction to correct for multiple comparisons (α ≤ .017). Our maximal models included *Condition* (control, accuracy feedback, narrative feedback), *Trial* (experimental, filler), and *Condition***Trial* interaction as fixed factors, and random intercepts and slopes for both subjects and items. The estimated coefficients, standard errors and the structure of the models are presented in Table 2.

In line with the findings of our mixed analysis of variance, the linear mixed effect analyses showed that participants thought addressees would perceive the speaker’s sarcasm more when their privileged information suggested that the speaker was being sarcastic (experimental trials) than when their privileged information suggested the speaker was being sincere (filler trials). More importantly, this difference between experimental and filler trials was significant in the control and accuracy feedback condition, but not in the narrative feedback condition. The findings of the *Condition***Trial* interactions showed a slightly different pattern than our mixed analysis of variance. In line with our previous findings, we found that participants in the control and accuracy feedback condition were to the same extent cursed by their privileged information, and that participants receiving narrative feedback were less biased by their privileged information than those in the control. However, whereas the mixed analysis of variance showed that participants receiving narrative feedback were less cursed by privileged information than those receiving accuracy feedback, the linear mixed effect analyses showed that this difference was less pronounced (i.e., only reached significance at the 95% CI).

| Table 1 *Estimated coefficients and standard errors for the mixed models (M1 to M4) fitted to readers’ judgment of addressees’ perception of sarcasm as a function of Condition (Control, Accuracy Feedback, Narrative Feedback) and Time (Time 1, Time 2)* | | | | | | | | |  |  |
| --- | --- | --- | --- | --- | --- | --- | --- | --- | --- | --- |
|  | *B* | | *SE b* | | *t* | | *99% CI* | |  |  |
| M1^1^ |  | |  | |  | |  | |  |  |
| Intercept (Control, Time 1) | 4.35 | | 0.15 | | 27.49 | | 3.96, 4.72 | |  |  |
| **Accuracy** **Feedback (Time 1)** | **-1.68** | | **0.15** | | **-9.65** | | **-2.07, -1.29** | |  |  |
| Narrative Feedback (Time 1) | -0.42 | | 0.16 | | -2.44 | | -0.84, 0.01* | |  |  |
| **Time 2 (Control)** | **-0.50** | | **0.13** | | **-3.88** | | **-0.83, -0.14** | |  |  |
| Accuracy Feedback * Time 2 | -0.38 | | 0.17 | | -2.06 | | -0.84, 0.04** | |  |  |
| **Narrative Feedback * Time 2** | **-1.84** | | **0.18** | | **-10.13** | | **[-2.33, -1.40]** | |  |  |
|  |  | |  | |  | |  | |  |  |
| M2^2^ |  | |  | |  | |  | |  |  |
| Intercept (Accuracy Feedback, Time 1) | 2.67 | | 0.15 | | 16.98 | | [2.31, 3.06] | |  |  |
| **Narrative Feedback (Time 1)** | **1.26** | | **0.17** | | **7.30** | | **[0.85, 1.71]** | |  |  |
| **Control (Time 1)** | **1.68** | | **0.15** | | **9.65** | | **[1.30, 2.09]** | |  |  |
| **Time 2** | **-0.88** | | **0.11** | | **-6.86** | | **[-1.18, -0.61]** | |  |  |
| **Narrative Feedback * Time 2** | **-1.46** | | **0.16** | | **-8.10** | | **[-1.87, -1.02]** | |  |  |
| Control * Time 2 | 0.38 | | 0.16 | | 2.06 | | [-0.04, 0.80]* | |  |  |
|  |  | |  | |  | |  | |  |  |
| M3^3^ |  | |  | |  | |  | |  |  |
| Intercept (Narrative Feedback, Time 2) | 1.58 | | 0.15 | | 10.14 | | [1.30, 1.89] | |  |  |
| Accuracy Feedback (Time 2) | 0.21 | | 0.18 | | 1.20 | | [-0.23, 0.67] | |  |  |
| **Control (Time 2)** | **2.26** | | **0.18** | | **13.07** | | **[1.83, 2.76]** | |  |  |
| **Time 1** | **2.34** | | **0.13** | | **18.45** | | **[2.02, 2.70]** | |  |  |
| **Accuracy Feedback * Time 1** | **-1.46** | | **0.20** | | **-8.10** | | **[-1.99, -0.98]** | |  |  |
| **Control * Time 1** | **-1.84** | | **0.18** | | **-10.13** | | **[-2.35, -1.42]** | |  |  |
|  |  | |  | |  | |  | |  |  |
| M4^4^ |  | |  | |  | |  | |  |  |
| Intercept (Control, Time 2) | 3.85 | | 0.16 | | 24.31 | | [3.44, 4.26] | |  |  |
| **Accuracy Feedback (Time 2)** | **-2.06** | | **0.15** | | **-11.81** | | **[-2.44, -1.65]** | |  |  |
| **Narrative Feedback (Time 2)** | **-2.26** | | **0.17** | | **-13.07** | | **[-2.68, -1.79]** | |  |  |
| **Time 1** | **0.50** | | **0.12** | | **3.88** | | **[0.20, 0.83]** | |  |  |
| Accuracy Feedback * Time 1 | 0.38 | | 0.18 | | 2.06 | | -0.10, 0.83* | |  |  |
| **Narrative Feedback * Time 1** | **1.84** | | **0.18** | | **10.13** | | **1.35, 2.27** | |  |  |
| *Notes*. Significant results are presented in bold. These results are based on the dataset in which outliers were excluded. Findings remained unchanged when the outliers were included into the analyses. Likelihood Ratio Tests were performed to check whether the maximal model that converged was significantly better than the random intercept model, and whether adding the order in which the items were presented to participants (*Order*) improved the models’ fit.  ^1^The control condition and time 1 were treated as reference categories. Only the random intercept model converged. Adding the order in which the items were presented to participants did improve the models’ fit, *χ^2^*(6) = 8.44, *p* = .207.*This comparison reached significance at the 95% CI [-0.74, -0.09]; **This comparison reached significance at the 95% CI [-0.74, -0.07].  ^2^The accuracy feedback condition and time 1 were treated as reference categories. Only the random intercept model converged. Adding the order in which the items were presented to participants did not improve the models’ fit, *χ^2^*(6) = 8.44, *p* = .207. *This comparison reached significance at the 95% CI [0.06, 0.70].  ^3^The narrative feedback condition time 2 were treated as reference categories. Only the random intercept model converged. Adding the order in which the items were presented to participants did not improve the models’ fit, *χ^2^*(6) = 8.44, *p* = .207.  ^4^ The control condition and time 2 were treated as reference categories. Adding the order in which the items were presented to participants did not improve the models’ fit, *χ^2^*(6) = 8.44, *p* = .207. *This comparison reached significance at the 95% CI [0.02, 0.72]. | | | | | | | | |  |  |
| Table 2 *Estimated coefficients and standard errors for the mixed models (M1 to M3) fitted to readers’ judgment of addressees’ perception of sarcasm as a function of Condition (Control, Accuracy Feedback, Narrative Feedback) and Trial (Experimental, Filler)* | | | | | | | | | | |
|  | | | *B* | | *SE b* | | *t* | | *99% CI* | |
| M1^1^ | | |  | |  | |  | |  | |
| Intercept (Control, Experimental) | | | 3.75 | | 0.12 | | 30.79 | | 3.45, 4.07 | |
| **Accuracy Feedback (Experimental)** | | | **-1.96** | | **0.15** | | **-12.19** | | **-2.36, -1.59** | |
| **Narrative Feedback (Experimental)** | | | **-2.17** | | **0.14** | | **-13.59** | | **-2.53, -1.80** | |
| **Filler (Control)** | | | **-0.64** | | **0.11** | | **-5.42** | | **-0.93, -0.36** | |
| Accuracy Feedback * Filler | | | 0.16 | | 0.14 | | 0.99 | | -0.18, 0.55 | |
| **Narrative Feedback * Filler** | | | **0.54** | | **0.16** | | **3.40** | | **0.14, 0.95** | |
|  | | |  | |  | |  | |  | |
| M2^2^ | | |  | |  | |  | |  | |
| Intercept (Accuracy Feedback, Experimental) | | | 1.79 | | 0.12 | | 14.64 | | 1.45, 2.09 | |
| Narrative Feedback (Experimental) | | | -0.01 | | 0.17 | | -1.31 | | -0.60, 0.27 | |
| **Control (Experimental)** | | | **1.96** | | **0.16** | | **12.19** | | **1.56, 2.37** | |
| **Filler** | | | **-0.48** | | **0.10** | | **-4.19** | | **-0.73, -0.20** | |
| Narrative Feedback * Filler | | | 0.39 | | 0.15 | | 2.38 | | -0.06, 0.73* | |
| Control * Filler | | | -0.16 | | 0.17 | | -0.99 | | -0.60, 0.26 | |
|  | | |  | |  | |  | |  | |
| M3^3^ | | |  | |  | |  | |  | |
| Intercept (Narrative Feedback, Experimental) | | | 1.58 | | 0.11 | | 13.03 | | 1.32, 1.88 | |
| Accuracy Feedback (Experimental) | | | 0.21 | | 0.15 | | 1.31 | | -0.18, 0.59 | |
| **Control (Experimental)** | | | **2.17** | | **0.15** | | **13.59** | | **1.78, 2.53** | |
| Filler | | | -0.10 | | 0.11 | | -0.89 | | -0.41, 0.15 | |
| Accuracy Feedback * Filler | | | -0.39 | | 0.15 | | -2.38 | | -0.74, 0.01** | |
| **Control * Filler** | | | **-0.54** | | **0.15** | | **-3.40** | | **-0.89, -0.13** | |
| *Notes*. Significant results are presented in bold. Likelihood Ratio Tests were performed to check whether the maximal model that converged was significantly better than the random intercept model, and whether adding the order in which the items were presented to participants (*Order*) improved the models’ fit.  ^1^The control condition and experimental trials were treated as reference categories. Adding both random intercepts and random slopes of *Trial* for both subjects and items did improve the model’s fit, *χ^2^*(4) = 18.04, *p* < .01, whereas adding *Order* to this maximal model did not, *χ^2^*(6) = 4.41 , *p* = .621  ^2^The accuracy feedback condition and experimental trials were treated as reference categories. Adding both random intercepts for subjects and items, and a random slope of *Trial* for subjects improved the model’s fit, *χ^2^*(2) = 17.54, *p* < .001, whereas adding *Order* to this maximal model did not, *χ^2^*(6) = 4.41, *p* = .622. *This comparison reached significance at the 95% CI [0.03, 0.63];  ^3^The narrative feedback condition and experimental trials were treated as reference categories. Adding both random intercepts for subjects and items, and a random slope of *Trial* for subjects improved the model’s fit, *χ^2^*(2) = 17.54, *p* < .001, whereas adding *Order* to this maximal model did not, *χ^2^*(6) = 4.41, *p* = .622. ***This comparison reached significance at the 95% CI [-0.65, -0.08]. | | | | | | | | | | |

**Learning Effects**

We explored the extent to which participants’ *final judgment* (for the last story) reflected the addressees’ actual perspective and whether this final score differed as a function of *Condition*. Recall that participants predicted addressees’ perception of the speaker’s sarcasm on a 7-point scale, whereby a score of 1 (i.e., definitely as sincere) reflected the addressees’ perspective. We computed a mean score of participants’ final judgment made on Time 2 and submitted this score to an one-way analysis of variance. The accuracy of participants’ final judgment differed as a function of *Condition*, *Brown-Forsythe F*(2, 95.56) = 36.01, *p* < .001. Planned contrast showed that participants’ final judgment in the accuracy feedback (*M* = 1.46, *SD* = 1.21) and narrative (*M* = 1.38, *SD* = 0.49) feedback conditions was more accurate than participants’ final judgment in the control condition (*M* = 3.18, *SD* = 1.48), *t*(60.54) = -7.30, *p* < .001. In addition, the accuracy of participants’ final judgment did not differ between the accuracy and narrative condition, *t*(59.29) = -0.38, *p* = .703.

As a final step in our exploratory analyses, we plotted participants’ first and second judgment of the addressees’ perspective over time, ranging from the first story participants read till the last (Figure 1). Visually inspection of the plots indicates that participants’ overall perspective-taking accuracy does not improve over time in the control condition. This learning effect, however, does seem to occur in the feedback conditions, especially in the condition in which participants received accuracy feedback about their perspective-taking accuracy. Participants’ first predictions of the addressees’ perspective seem to have benefitted the most from the accuracy feedback, allowing participants to increase their accuracy over time. This in contrast to the narrative feedback condition, in which participants relied most on privileged information when *first* predicting addressees’ perspective to the same degree over time, and only adjusted their predictions after they received addressees’ uptake of the speaker’s message. Interestingly, this uptake did not seem to influence participants’ learning effect over time, as their accuracy on first predictions did not improve over time.

However, an important question that arises here is whether the increase of accuracy on Time 1 in the accuracy feedback condition constitutes a true learning effect. As we have previously seen, the accuracy scores of participants’ second judgments did not differ between the two feedback conditions, nor did the accuracy of their final judgments (final story). Moreover importantly, however, we showed that, regardless of this increase in accuracy on Time 1, participants in the accuracy feedback condition still misattributed their perception of sarcasm on Time 2, whereas participants in the narrative feedback condition did not (see section “Curse of Knowledge Effect and Feedback” in the main article). Hence, these findings seem to suggest that accuracy feedback might only slightly increase participants’ accuracy because they learn how the task should be performed. Based on our findings, however, we suggest here that accuracy feedback does not suffice to reduce participants’ egocentric projection and, as a result, their interpersonal accuracy.

All findings remain unchanged when the outliers were included into the analyses and when we controlled for the order in which the scenarios appeared in the booklets (normal, reversed). Considering the gender imbalance in our design, we also controlled for possible gender differences. Results showed that all findings remained unchanged and that the main effect of *Gender*, *F*(1, 133) = 0.03, *p* = .860, as well as the *Gender***Time* interaction, *F*(1, 133) = 0.27, *p* = .604, on perspective-taking accuracy were both non-significant.


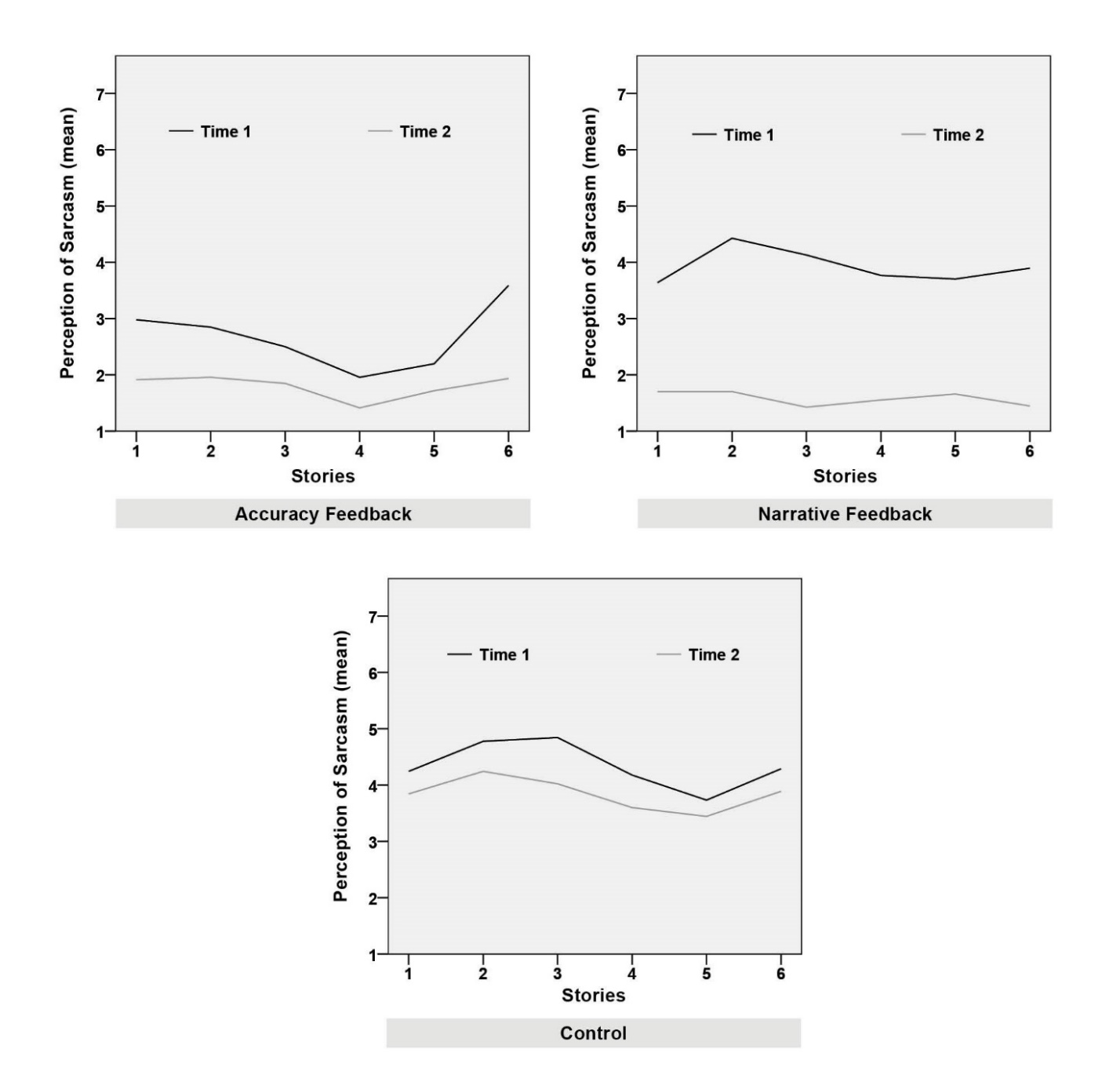


*Figure 1*. Participants’ first (Time 1) and second judgment (Time 2) of addressees’ perception of sarcasm (1 = definitely as sincere, 7 = definitely as sarcastic; mean score) plotted over time for the 6 stories communicating a Experimental Trial for the accuracy feedback, narrative feedback and control condition.

**Participants’ Perspective-Taking Tendency**

We explored the relationship between participants' self-reported perspective-taking tendency and their actual perspective-taking behavior. As part of this exploratory analysis, we first investigated whether participants’ propensity to engage in perspective-taking (self-report) differed as a function of condition. Subsequently, we examined the extent to which this self-reported propensity predicted participants’ perspective-taking accuracy during the experiment. Exploratory analyses revealed three additional outliers in the narrative feedback condition and one additional outlier in the control condition (the deviance ranged from 1.87 to 2.3). After excluding these outliers, normality improved and participants’ self-reported perspective-taking tendency was normally distributed in the accuracy feedback (*Z_skewness_* = -0.97, *Z_kurtosis_* = -1.21), narrative feedback (*Z_skewness_* = -0.33, *Z_kurtosis_* = -0.47), and control condition (*Z_skewness_* = 1.44, *Z_kurtosis_* = -0.70). A one-way analysis revealed that participants’ perspective-taking tendency did not differ between conditions, *Welch’s F*(2, 85.69) = 0.33, *p* = .717. Participants in the accuracy feedback (*M* = 4.18, *SD* = .87), narrative feedback (*M* = 4.30, *SD* = .67), and control (*M* = 4.22, *SD* = 0.61) condition reported to have regarded the addressee protagonists’ perspective during the experiment to the same degree. Findings remained unchanged when the outliers were included into the analysis.

In a follow-up linear mixed effect analysis, we investigated whether participants’ self-reported perspective-taking predicted the accuracy of their second perspective-judgment. We created a full model that included participants’ self-report as a fixed effect and random intercepts for both subjects and items. We obtained the *p*-values using the Likelihood Ratio Test (LRT). In this test, we compared the full model with the intercept only model. This LRT test revealed that participants’ self-report did not predict their perspective-taking accuracy, *χ^2^*(1) = 0.87, *p* = .352; *b* = -0.01, *SE_b_* = 0.02, *t* = -0.93. Findings remained unchanged when the outliers were included into the analysis.

**Experiment 2**

**Linear Mixed Effects Analyses**

**Feedback and Perspective Adjustments.** We construed two maximal models that included *Time* *Measurement* (one-shot, two-shot), *Narrative Feedback* (absent, present), and *Time Measurment***Narrative Feedback* interaction as fixed factors, and random intercepts and slopes for both subjects and items. We estimated the confidence intervals and *p*-values by parametric bootstrapping over 100 iterations, and employed the Bonferroni correction for multiple comparisons (α ≤ .025). The estimated coefficients, standard errors and the structure of the models are presented in Table 3.

The results of the linear mixed effects analyses replicated those found in our mixed analysis of variance. We found a significant main effect of *Narrative Feedback* on participants’ attribution of sarcasm, whereas the main effect of *Time Measurement*, and the *Time Measurement*Narrative Feedback* interaction were non-significant. Participants attributed the perception of sarcasm onto addressees more when narrative feedback was absent than when it was present. Furthermore, participants’ attribution of sarcasm did not depend on them judging addressees’ perspective once (one-shot) or twice (two-shot) for each scenario, and this pattern remained the same whether participants did or did not receive narrative feedback.

**Curse of Knowledge Effect**. We construed two maximal models that included *Narrative Feedback* (absent, present) and *Trial* (experimental, filler), and *Narrative Feedback***Trial* interaction as fixed factors, and random intercepts and slopes for both subjects and items. We estimated the confidence intervals and *p*-values by parametric bootstrapping over 100 iterations, and employed the Bonferroni correction for multiple comparisons (α ≤ .025). The estimated coefficients, standard errors and the structure of the models are presented in Table 4.

The results of the linear mixed effects analyses replicated those found in our mixed analysis of variance. We found a main effect of *Trial* (experimental, filler) that was qualified by a significant interaction with *Narrative Feedback* (absent, present). More specifically, when narrative feedback was absent, participants were more likely to attribute their perception of the speaker’s sarcasm onto addressees when their privileged information suggested that the speaker was being sarcastic (experimental trials) than when participants’ privileged information suggested the speaker was being sincere (filler trials). When participants did receive narrative feedback this difference in participants’ attribution of the speaker’s sarcasm between experimental and filler trials disappeared.

| Table 3 *Estimated coefficients and standard errors for the mixed models (M1, M2) fitted to readers’ judgment of addressees’ perception of sarcasm as a function of Narrative Feedback (Absent, Present) Time Measurement (One-Shot, Two-Shot)* | | | | |
| --- | --- | --- | --- | --- |
|  | *B* | *SE b* | *t* | *99% CI* |
| M1^1^ |  |  |  |  |
| Intercept (Absent, One-Shot) | 3.66 | 0.16 | 22.84 | 3.26, 4.10 |
| Two-Shot (Absent) | -0.23 | 0.13 | -1.98 | -0.57, 0.08 |
| **Present (One-Shot)** | **-2.11** | **0.18** | **-12.41** | **-2.61, -1.66** |
| Present * Two-Shot | 0.14 | 0.17 | 0.83 | -2.74, -1.52 |
|  |  |  |  |  |
| M2^2^ |  |  |  |  |
| Intercept (Present, Two-Shot) | 1.46 | 0.11 | 12.93 | 1.16, 1.74 |
| One-Shot (Present) | 0.09 | 0.11 | 0.81 | -0.18, 0.37 |
| **Absent (Two-Shot)** | **1.97** | **0.12** | **16.99** | **1.67, 2.27** |
| Absent * One-Shot | 0.14 | 0.15 | 0.84 | -0.24, 0.51 |
| *Notes*. Significant results are presented in bold. Findings remained unchanged when the outlier was included into the analyses. Likelihood Ratio Tests were performed to check whether the maximal model that converged was significantly better than the random intercept model, and whether adding the order in which the items were presented to participants (*Order*) improved the models’ fit.  ^1^The one-shot and narrative feedback absent conditions were treated as reference categories. Compared to the random intercept model, adding random intercepts for subjects and items, as well as random slopes of *Narrative Feedback* for items improved the model’s fit, *χ^2^*(2) = 46.22, *p* < .001. Adding *Order* to this maximal model did not improve the model’s fit, *χ^2^*(4) = 4.97 , *p* = .291  ^2^The two-shot and narrative feedback present conditions were treated as reference categories. Only the random intercept model reach convergence. Compared to this model, adding the order in which the items were presented to participants did not improve the models’ fit, *χ^2^*(1) = 0.41 , *p* = .524 | | | | |

| Table 4 *Estimated coefficients and standard errors for the mixed models (M1, M2) fitted to readers’ judgment of addressees’ perception of sarcasm as a function of Narrative Feedback (Absent, Present) and Trial (Experimental, Filler)* | | | | |
| --- | --- | --- | --- | --- |
|  | *B* | *SE b* | *t* | *99% CI* |
| M1^1^ |  |  |  |  |
| Intercept (Absent, Experimental) | 3.93 | 0.15 | 24.90 | 3.56, 4.32 |
| **Present (Experimental)** | **-2.46** | **0.17** | **-13.87** | **-2.92, -2.02** |
| **Filler (Absent)** | **-0.79** | **0.09** | **-8.31** | **-1.03, -0.55** |
| **Present * Filler** | **0.74** | **0.13** | **5.33** | **0.40, 1.09** |
|  |  |  |  |  |
| M2^2^ |  |  |  |  |
| **Intercept (Present, Filler)** | **1.42** | **0.09** | **17.96** | **1.19, 1.66** |
| **Absent (Filler)** | **1.72** | **0.19** | **10.09** | **1.28, 2.24** |
| Experimental (Present) | 0.05 | 0.10 | 0.46 | -0.22, 0.32 |
| **Absent * Experimental** | **0.74** | **0.14** | **5.33** | **0.36, 1.08** |
| *Notes*. Significant results are presented in bold. These results are based on the dataset in which outliers were excluded. Findings remained unchanged when the outliers were included into the analyses. Likelihood Ratio Tests were performed to check whether the maximal model that converged was significantly better than the random intercept model, and whether adding the order in which the items were presented to participants (*Order*) improved the models’ fit.  ^1^The narrative feedback absent and experiment were treated as reference categories. Compared to the random intercept model, adding both random intercepts and random slopes of *Trial* for both subjects and items did improve the model’s fit, *χ^2^*(4) = 18.04, *p* < .01, whereas adding *Order* did not, *χ^2^*(6) = 4.41 , *p* = .621  ^2^The narrative feedback present and filler trials were treated as reference categories. Random intercepts for both subjects and items, as well as random slopes of *Trial* for subjects and a random slope of *Narrative Feedback* for items improved the model’s fit, *χ^2^*(4) = 84.23, *p* < .001. Adding *Order* to this maximal model did not improve the models’ fit, *χ^2^*(4) = 3.18, *p* = .529. | | | | |

### Participants’ Perspective-Taking Tendency

As in Experiment 1, we explored the relationship between participants' self-reported perspective-taking tendency and their actual perspective-taking behavior. First, we examined whether participants’ propensity to engage in perspective-taking (self-report) differed as a function of condition. In particular, we tested whether participants’ perspective-taking tendency differed between the two new one-shot conditions (one-shot control, one-shot narrative feedback), and whether the two new one-shot conditions differed from the two-shot conditions (one-shot control vs. two-shot control, one-shot narrative feedback vs. two-shot narrative feedback). Following our preregistration, we excluded additional outliers to improve the normal distribution of the data. Participants’ perspective-taking tendency was normally distributed after excluding one outlier in the one-shot control condition (deviance = 2.01), two outliers in the two-shot narrative feedback condition (deviance = 1.95, 2.32) and one outlier in the two-shot control condition (deviance = 1.86)^^[[1]](#footnote-1)^^. A one-way analysis revealed that participants’ tendency to engage in perspective-taking significantly differed between conditions, *F*(3, 176) = 8.38, *p* < .001. Planned contrasts revealed that participants in the one-shot narrative feedback condition (*M* = 4.90, *SD* = 0.87) reported a higher perspective-taking tendency than participants in the one-shot control condition (*M* = 4.32, *SD* = 0.74), *t*(176) = -3.72, *p* < .001. The second planned contrast showed that there we no significant differences between the one-shot control and the two-shot control condition (*M* = 4.23, *SD* = 0.62), *t*(176) = -0.59, *p* = .555. However, the last planned contrast showed that participants in the one-shot narrative feedback condition did report a higher perspective-taking tendency than the participants in the two-shot narrative feedback condition (*M* = 4.28, *SD* = 0.67), *t*(176) = -3.99, *p* < .001. All findings remained unchanged when the outliers were included into the analyses.

In a follow-up linear mixed effect analysis, we investigated whether participants’ self-reported perspective-taking predicted the accuracy of their judgment. We created a full model that included participants’ self-report as a fixed effect and random intercepts for both subjects and items. We obtained the *p*-values using the Likelihood Ratio Test (LRT). In this test, we compared the full model with the intercept only model. This LRT test revealed that participants’ self-report predicted their perspective-taking accuracy, *χ^2^*(1) = 15.08, *p* < .001; *b* = -0.05, *SE_b_* = 0.01, *t* = -3.97. Findings remained unchanged when the outliers were included into the analysis.

**References**

Baayen, R. H., Davidson, D. J., & Bates, D. M. (2008). Mixed-effects modeling with crossed
 random effects for subjects and items. *Journal of Memory and Language, 59*(4), 390–
 412. <https://doi.org/10.1016/j.jml.2007.12.005>

Baayen, H. R., & Milin, P. (2010). Analyzing reaction times. *International Journal of
 Psychological Research, 3*(2), 12. <https://doi.org/10.21500/20112084.807>

Barr, D. J., Levy, R., Scheepers, C., & Tily, H. J. (2013). Random effects structure for
 confirmatory hypothesis testing: Keep it maximal. *Journal of Memory and Language, 68*,
 255–278

Bates, D., Mächler, M., Bolker, B. M., & Walker, S. C. (2015). Fitting Linear Mixed-Effects
 Models using lme4. *Journal of Statistical Software, 67*(1).
 <https://doi.org/10.18637/jss.v067.i01>

R Core Team. (2017). R: A language and environment for statistical computing. Vienna: R
 Foundation for Statistical Computing.

1. Participants’ self-reported perspective-taking tendency was normally distributed in the one-shot control (*Z_skewness_* = 0.72, *Z_kurtosis_* = 0.54), one-shot narrative feedback (*Z_skewness_* = 1.40, *Z_kurtosis_* = -0.10), the two-shot control (*Z_skewness_* = 1.46, *Z_kurtosis_* = -0.83), and the two-shot narrative feedback condition (*Z_skewness_* = -0.20, *Z_kurtosis_* = -0.59). [↑](#footnote-ref-1)
